# Detailed Modeling of the Direct Reduction of Iron Ore in a Shaft Furnace

**DOI:** 10.3390/ma11101865

**Published:** 2018-10-01

**Authors:** Hamzeh Hamadeh, Olivier Mirgaux, Fabrice Patisson

**Affiliations:** 1 Institut Jean Lamour, CNRS, Université de Lorraine, 54011 Nancy, France; hamzeh.hamadeh@univ-lorraine.fr (H.H.); olivier.mirgaux@univ-lorraine.fr (O.M.); 2 Laboratory of Excellence on Design of Alloy Metals for Low-Mass Structures (DAMAS), Université de Lorraine, 57073 Metz, France

**Keywords:** ironmaking, direct reduction, iron ore, DRI, shaft furnace, mathematical model, heterogeneous kinetics, heat and mass transfer

## Abstract

This paper addresses the modeling of the iron ore direct reduction process, a process likely to reduce CO_2_ emissions from the steel industry. The shaft furnace is divided into three sections (reduction, transition, and cooling), and the model is two-dimensional (cylindrical geometry for the upper sections and conical geometry for the lower one), to correctly describe the lateral gas feed and cooling gas outlet. This model relies on a detailed description of the main physical–chemical and thermal phenomena, using a multi-scale approach. The moving bed is assumed to be comprised of pellets of grains and crystallites. We also take into account eight heterogeneous and two homogeneous chemical reactions. The local mass, energy, and momentum balances are numerically solved, using the finite volume method. This model was successfully validated by simulating the shaft furnaces of two direct reduction plants of different capacities. The calculated results reveal the detailed interior behavior of the shaft furnace operation. Eight different zones can be distinguished, according to their predominant thermal and reaction characteristics. An important finding is the presence of a central zone of lesser temperature and conversion.

## 1. Introduction

**Figure 1:**
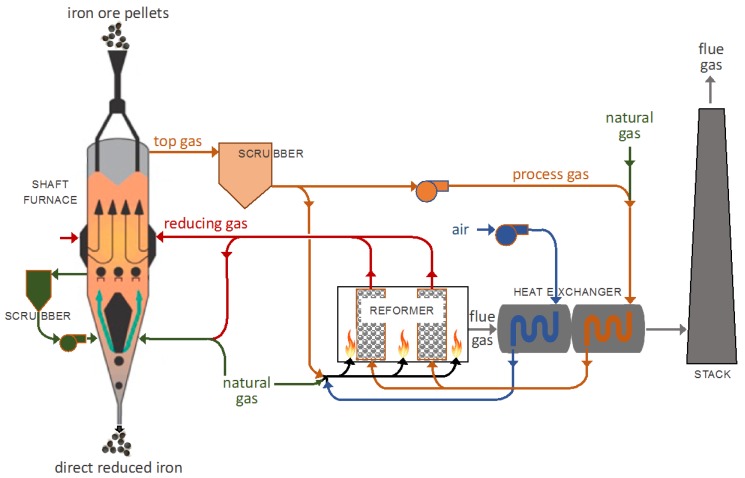
MIDREX process flowsheet.

The direct reduction (DR) of iron ore, usually followed by electric arc steelmaking, is an alternative route to the standard, blast furnace, basic oxygen route for making steel. Annual DR iron production (86 Mt in 2017) remains small, compared to the production of 1180 Mt of blast furnace pig iron [1]. However, an attractive feature of DR, compared to blast furnace reduction, is its considerably lower CO_2_ emissions, which are 40 to 60% lower for the DR-electric arc furnace route, compared to the blast furnace, basic oxygen route [2]. Among DR processes, shaft furnaces represent over 82% of the world’s DR iron production, with the two main processes being MIDREX (65%), as shown in Figure 1, and HYL-ENERGIRON (17%) [3].

In a DR shaft furnace, a charge of pelletized or lump iron ore is loaded into the top of the furnace and is allowed to descend, by gravity, through a reducing gas. The reducing gas, comprised of hydrogen and carbon monoxide (syngas), and obtained by the catalytic reforming of natural gas, flows upwards, through the ore bed. Reduction of the iron oxides occurs in the upper section of the furnace, at temperatures up to 950 °C. A transition section is found below the reduction section; this section is of sufficient length to separate the reduction section from the cooling section, allowing an independent control of both sections. The solid product, called direct reduced iron (DRI) or reduced sponge iron, is cooled in the lower part of the furnace, down to approximately 50 °C, prior to being discharged.

The modeling of a shaft furnace, simulating the reduction of iron ore by syngas, is a powerful tool for defining optimal operating conditions. Use of such a model can lead to the maximization of conversion or the minimization of energy consumption, among other effects capable of reducing carbon dioxide emissions. As such, numerous iron ore shaft furnace models have been proposed in the literature. Initial studies addressed the reduction of a single pellet by H_2_, CO, or H_2_-CO mixtures [4,5,6,7,8,9]. Subsequent studies developed models that simulated the reduction zone of the shaft furnace in one dimension [10,11]. With the aim of correctly describing the lateral gas feed, some studies have introduced two-dimensional models [12,13,14]; however, these models did not consider the presence of methane, which is responsible for important reactions in the process. More recently, several authors introduced other reactions [15] and accounted for the cooling zone [16,17]. Some even developed plant models [18]; however, these works were limited to one-dimensional models.

In this work, we developed further the model of Ranzani Da Costa and Wagner, built to simulate the reduction section of DR shafts, operated with pure hydrogen [13,14,19]. We extended this model to consider CO-H_2_-CH_4_ reducing gas, and accounted for transition and cooling sections. The present model, named REDUCTOR, is 2-dimensional in the steady-state regime. The model includes a sophisticated, pellet sub-model. We consider eight heterogeneous and two homogeneous chemical reactions. These features represent a more advanced and detailed model, compared to previous studies. Moreover, the results were validated against two sets of plant data.

The present model, REDUCTOR, differs from the other model we recently reported [18] on the following points. REDUCTOR is a computational fluid dynamics (CFD)-type, two-dimensional model, which describes the shaft furnace alone. The other model is of the systemic type, is one-dimensional, and aims to simulate the whole DR plant. The shaft furnace description included in the plant model, though based on similar equations, was intentionally made simpler and faster to run, on process simulation software. Thus, REDUCTOR is more detailed and more precise, but, of course, requires longer computation times.

## 2. Mathematical Model

### 2.1. Principle

The reduction of hematite ore to iron occurs via two intermediate oxides, namely, magnetite and wüstite (considered as Fe_0.95_O [19]), and by two gaseous reactants, namely, H_2_ and CO. The following six reduction reactions were therefore considered:

(1) $$\;3{\mathrm{Fe}}_{2}O_{3}{}_{\left(s\right)}+H_{2}{}_{\left(g\right)}\to 2{\mathrm{Fe}}_{3}O_{4}{}_{\left(s\right)}+H_{2}O_{\left(g\right)}\;$$

(2) $$\;{\mathrm{Fe}}_{3}O_{4}{}_{\left(s\right)}+\frac{16}{19}H_{2\left(g\right)}\to \frac{60}{19}{\mathrm{Fe}}_{0.95}O_{\left(s\right)}+\frac{16}{19}H_{2}O_{\left(g\right)}\;$$

(3) $$\;{\mathrm{Fe}}_{0.95}O_{\left(s\right)}+H_{2}{}_{\left(g\right)}\to 0.95{\mathrm{Fe}}_{\left(s\right)}+H_{2}O_{\left(g\right)}\;$$

(4) $$\;3{\mathrm{Fe}}_{2}O_{3}{}_{\left(s\right)}+{\mathrm{CO}}_{\left(g\right)}\to 2{\mathrm{Fe}}_{3}O_{4}{}_{\left(s\right)}+{\mathrm{CO}}_{2}{}_{\left(g\right)}\;$$

(5) $$\;{\mathrm{Fe}}_{3}O_{4}{}_{\left(s\right)}+\frac{16}{19}{\mathrm{CO}}_{\left(g\right)}\to \frac{60}{19}{\mathrm{Fe}}_{0.95}O_{\left(s\right)}+\frac{16}{19}{\mathrm{CO}}_{2}{}_{\left(g\right)}\;$$

(6) $$\;{\mathrm{Fe}}_{0.95}O_{\left(s\right)}+{\mathrm{CO}}_{\left(g\right)}\to 0.95{\mathrm{Fe}}_{\left(s\right)}+{\mathrm{CO}}_{2}{}_{\left(g\right)}\;$$

Methane reforming and water gas shift reactions also occur in the gas phase, based on the composition of reduction gas and temperature, through the following reactions:

(7) $$\;{\mathrm{CH}}_{4\left(g\right)}+H_{2}O_{\left(g\right)}⇌{\mathrm{CO}}_{\left(g\right)}+3H_{2\left(g\right)}\;$$

(8) $$\;{\mathrm{CO}}_{\left(g\right)}+H_{2}O_{\left(g\right)}⇌{\mathrm{CO}}_{2\left(g\right)}+H_{2\left(g\right)}\;$$

We also considered two other side reactions that could occur in the reactor, especially where an iron layer has formed:

- Methane decomposition reaction
  (9) $$\;{\mathrm{CH}}_{4\left(g\right)}⇌C_{\left(s\right)}+2H_{2\left(g\right)}\;$$
- Carbon monoxide disproportionation (inverse Boudouard reaction)
  (10) $$\;2{\mathrm{CO}}_{\left(g\right)}\;⇌C_{\left(s\right)}+{\mathrm{CO}}_{2\left(g\right)}\;$$

**Figure 2:**
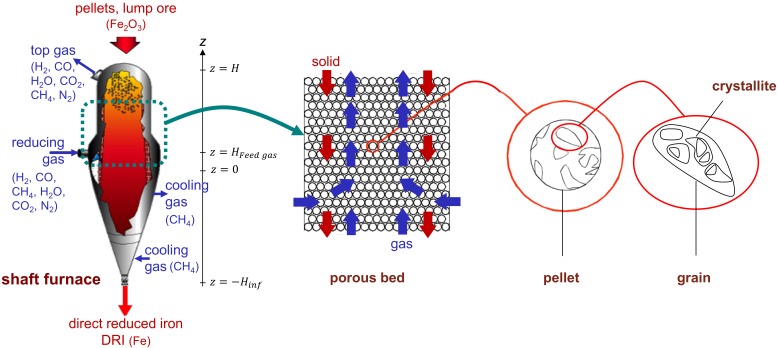
Schematic representation of the REDUCTOR model, from the reactor scale to the crystallite scale (see Appendix A for notations).

The model itself is two-dimensional, axisymmetrical, and steady-state. It is based on the numerical solution of local mass, energy, and momentum balances, using the finite volume method. The geometry in the reduction and transition sections is cylindrical, while conical in the cooling section. This corresponds to the geometry of the shaft furnaces and is necessary to describe correctly the lateral gas feed and outlet cooling gas, as shown in Figure 2. The reactor modeled is a shaft furnace of the MIDREX type.

The solid load is fed from the top of the reactor (z=H) to form a moving bed of solid particles composed of spherical iron ore pellets that descend by gravity. The pellet diameter (d_p_) is assumed to be unique and unchanging during the reduction reaction, and the initial pellet composition is known. The gas phase is composed of six species: H_2_, CO, H_2_O, CO_2_, N_2_, and CH_4_. The reducing gas is injected from the sidewall, at a height of $z=H_{\mathrm{Feed},\mathrm{gas}}\;$which then moves upward, against the solid flow, before finally exiting the furnace at the top. The temperature and composition of this reducing gas are known. A secondary feed gas—the cooling gas—which is introduced from the bottom of the furnace ($z=-H_{\inf}$), is also considered. This cooling gas exits the furnace, through the wall in the upper part of the conical section. The temperatures of the solid and gas are different and vary, according to their position (r, z) within the furnace. The solid temperature is assumed to be uniform in the interior of the pellets. Thus, this model is based on a faithful description of the physical-chemical and thermal phenomena, from the reactor scale to the crystallite scale, as shown in Figure 2. In the pellet sub-model, the pellet is assumed to be initially comprised of dense grains; these grains later fragment into smaller crystallites at the wüstite stage, in agreement with microscopic observations [19]. Thus, from the reactor to the crystallites, we have a 4-scale model.

### 2.2. Equations

#### 2.2.1. Gas Phase

The descending solid pellets, through which the ascending gas flows, can be considered a porous medium, consisting of quasi-stationary solid spheres (the gas velocity is much greater than that of the solid). The Ergun equation (see Appendix A for nomenclature), combined with the continuity equation, thus gives

(11) $$\;\frac{1}{r}\frac{\partial }{\partial r}\left(\frac{rc_{t}}{K}\frac{\partial p}{\partial r}\right)+\frac{\partial }{\partial z}\left(\frac{c_{t}}{K}\frac{\partial p}{\partial z}\right)=S_{mol,tot}=2v_{7}+v_{9}-v_{10}\;$$

where the terms are in units, ${\mathrm{mol}\;m}^{-3}s^{-1}$; *K* is the permeability coefficient, calculated as

(12) $$\;K=\frac{150{\left(1-\varepsilon_{b}\right)}^{2}}{\varepsilon_{b}^{3}d_{p}^{2}}{µ}_{g}+\frac{1.75\left(1-\varepsilon \right)}{\varepsilon_{b}^{3}d_{p}}\rho_{g}u_{g}\;$$

and the source term $S_{mol,tot}$ corresponds to the net gas production by the non-equimolar reactions. Equation (11) is used to calculate the pressure field, and the gas velocity vector is calculated, using Equation (13):

(13) $$u_{g}=-\frac{1}{K}\nabla P$$

The mass balance for a gaseous species, *i*, considering axial and radial dispersion, in addition to convection, is written:

(14) $$\;\frac{1}{r}\frac{\partial \left(rc_{t}x_{i}u_{g,r}\right)}{\partial r}+\frac{\partial \left(c_{t}x_{i}u_{g,z}\right)}{\partial z}=\frac{1}{r}\frac{\partial }{\partial r}\left(rc_{t}D_{r}\frac{\partial x_{i}}{\partial r}\right)+\frac{\partial }{\partial z}\left(c_{t}D_{z}\frac{\partial x_{i}}{\partial z}\right)+S_{i}\;$$

with the source term, *S_i_*, given in Table 1.

The heat balance for the gas phase—considering convection and conduction—as well as the heat exchanged with the solid and heat brought by the gases evolving from the solid, gives:

(15) $$\;\rho_{g}c_{pg}\left(u_{gr}\frac{\partial T_{g}}{\partial r}+u_{gz}\frac{\partial T_{g}}{\partial z}\right)=\frac{1}{r}\frac{\partial }{\partial r}\left(r\lambda_{g}\frac{\partial T_{g}}{\partial r}\right)+\frac{\partial }{\partial z}\left(\lambda_{g}\frac{\partial T_{g}}{\partial z}\right)+a_{b}h\left(T_{s}-T_{g}\right)+\sum_{i}S_{i}\int_{T_{g}}^{T_{s}}c_{p}{}_{i}dT\;$$

#### 2.2.2. Solid Phase

Regarding the grain flow, in the upper cylindrical section, it is considered that pellets descend vertically. In contrast, in the lower section of the conical shape, a radial component of the solid velocity must be introduced. A bibliographical study of granular flows led us to use the model of Mullins [20,21], in which the radial velocity is calculated as proportional to the radial gradient of the axial velocity:

(16) $$\;u_{s,r}=-B\frac{\partial u_{s,z}}{\partial r}\;$$

where *B* is taken, as proposed in Reference [20]:

(17) $$\;B=2d_{p}\;$$

The mass balance for a gaseous species *j* gives:

(18) $$\;-\frac{\partial \left(\rho_{b}u_{s,z}w_{j}\right)}{\partial z}+\frac{1}{r}\frac{\partial \left(r\rho_{b}u_{s,r}w_{j}\right)}{\partial r}=S_{j}\;$$

with the source term, *S_j_*, given in Table 2.

The heat balance for the solid phase takes into account axial and radial convection, conduction, and heat exchange with the gas phase. The heat of the reactions is attributable to the solid phase, considering that all the reactions occur either inside the pellets (heterogeneous reactions) or at their surfaces (homogeneous reactions, catalyzed by the solid); thus:

(19) $$\;-\rho_{b}u_{s,z}c_{ps}\frac{\partial T_{s}}{\partial z}+\rho_{b}u_{s,r}c_{ps}\frac{\partial T_{s}}{\partial r}=\frac{1}{r}\frac{\partial }{\partial r}\left(r\lambda_{eff,r}\frac{\partial T_{s}}{\partial r}\right)+\frac{\partial }{\partial z}\left(\lambda_{eff,z}\frac{\partial T_{s}}{\partial z}\right)+a_{b}h\left(T_{g}-T_{s}\right)+\sum_{n=1}^{10}(-v_{n}\Delta_{r}H_{n})\;$$

### 2.3. Transport Coefficients

The various transport coefficients, $D_{r},\;D_{z},\;\lambda_{eff,r},\;\mathrm{and}\;\lambda_{eff,z}$, as well as other parameters, like specific heats, are calculated as functions of temperature and composition. Details regarding the relationships were given in [22].

### 2.4. Reaction Rates

#### 2.4.1. Iron Oxide Reduction

Unlike most of the previous approaches published which are based on the shrinking core model (with one or three fronts, separating the oxides in the pellet), we developed a specific, pellet sub-model. The sub-model was built, according to our experimental findings, to simulate the reduction of a single pellet by H_2_-CO. The reaction rate was used as a function of the local reduction conditions (temperature and gas composition), inside the reactor. We used the law of additive reaction times [23], which considers the different resistances (chemical reaction, diffusion, external transfer) involved in series. Therefore, the time required to attain a certain conversion is approximately the sum of the characteristic times: $\tau_{i}$ [14,23]. This sub-model was initially developed for simulating reduction by H_2_ only, as detailed previously [14]; we extended this model for reduction by CO. The characteristic times and the reaction rates are listed in Appendix B.

#### 2.4.2. Methane Reforming and Water Gas Shift Reactions

Methane reforming and water gas shift reactions are known to be catalyzed by iron or iron oxides [24,25]; thus, their rates are functions of the composition of the reduction gas, temperature, and mass of the catalyst. The methane reforming rate equation considering the forward and reverse reactions is given by Equation (20):

(20) $$\;v_{7}=k_{7}\left(1-\varepsilon_{b}\right)\left(1-\varepsilon_{interg}\right)\left(P_{{\mathrm{CH}}_{4}}P_{H_{2}O}-\frac{P_{\mathrm{CO}}P_{H_{2}}^{3}}{K_{eq,7}}\right)\;$$

The expression of the reaction rate constant, *k*_7_, is given in Table 3. Because the reforming of CH_4_ was hardly observed on the iron oxide catalysts, as reported in the literature [25], it was considered that such reforming only occurs with iron as a catalyst. We assumed that sufficient iron was formed on the outside of the pellet, when the reduction degree exceeded 50%.

Similarly, the rate expression for the water gas shift reaction is given by Equation (21):

(21) $$\;v_{8}=k_{8}\left(1-\varepsilon_{b}\right)\left(1-\varepsilon_{interg}\right)\left(P_{\mathrm{CO}}P_{H_{2}O}-\frac{P_{{\mathrm{CO}}_{2}}P_{H_{2}}}{K_{eq,8}}\right)\;$$

when occurring on Fe or Fe_0.95_O, and by Equation (22)

(22) $$\;v_{8}=k_{8}^{\prime }\rho_{c}\left(1-\varepsilon_{b}\right)\left(1-\varepsilon_{interg}\right)\left(P_{\mathrm{CO}}P_{H_{2}O}-\frac{P_{{\mathrm{CO}}_{2}}P_{H_{2}}}{K_{eq,8}}\right)\;$$

when occurring on Fe_2_O_3_ or Fe_3_O_4_. Here, besides iron, various iron oxides also catalyze the reaction. The corresponding expressions for $k_{8}$ and $k_{8}^{\prime }$ are given in Table 3, according the literature [24,25].

#### 2.4.3. Carbonization Reactions

In the DR furnace, carbon can be formed, either from methane decomposition (Equation (9)) or from CO disproportionation (Equation (10)). Both reactions are reversible, and the reverse reactions are functions of the carbon activity. The carbon activity was calculated from Chipman’s relationship [27]:

(23) $$\;\log a_{c}=\frac{2300}{T}-0.92+\left(\frac{3860}{T}\right)C+\log\left(\frac{C}{1-C}\right)\;$$

where *C* is the ratio of atomic C to atomic Fe. For sake of simplicity, we did not distinguish between C and Fe_3_C in the solid, with both being considered as C.

The rate equation of the methane decomposition reaction is given by Equation (24)

(24) $$\;v_{9}=\frac{k_{9}}{P_{H_{2}}^{0.5}}\left(1-\varepsilon_{b}\right)\left(1-\varepsilon_{interg}\right)\left(P_{{\mathrm{CH}}_{4}}-\frac{P_{H_{2}}^{2}a_{c}}{K_{eq,9}}\right)\;$$

The expression of the reaction rate constant, $k_{9}$, included in Equation (24) was determined, as per the literature [16,26], as listed in Table 3.

The rate equation of the carbon monoxide disproportionation reaction is given by Equation (25)

(25) $$\;v_{10}=\left(k_{10}P_{H_{2}}^{0.5}+k_{10}^{\prime }\right)\left(1-\varepsilon_{b}\right)\left(1-\varepsilon_{interg}\right)\left(P_{\mathrm{CO}}^{2}-\frac{P_{{\mathrm{CO}}_{2}}a_{c}}{K_{eq,10}}\right)\;$$

and the values of the reaction rate constants, $k_{10}\;$and $k_{10}^{\prime }$, are also provided in Table 3, from the same references.

### 2.5. Boundary Conditions

The balance equations need a set of associated boundary conditions to be solved. First, the temperature and composition of the solids and gases are assumed to be known (the operating conditions) at their respective inlets (at the top for solids, and at the bottom and sides for gases). In addition, because of axisymmetry and tight walls, one has:

(26) $$-\;\mathrm{Symmetry}\;\mathrm{axis}:\;\mathrm{zero}\;\mathrm{fluxes}\;\frac{\partial T_{s}}{\partial r}=\frac{\partial T_{g}}{\partial r}=\frac{\partial x_{i}}{\partial r}=0\;-\;\mathrm{Side}\;\mathrm{wall}\;(\mathrm{except}\;\mathrm{gas}\;\mathrm{inlet}):\;\frac{\partial T_{s}}{\partial r}=\frac{\partial T_{g}}{\partial r}=\frac{\partial x_{i}}{\partial r}=0$$

**Figure 3:**
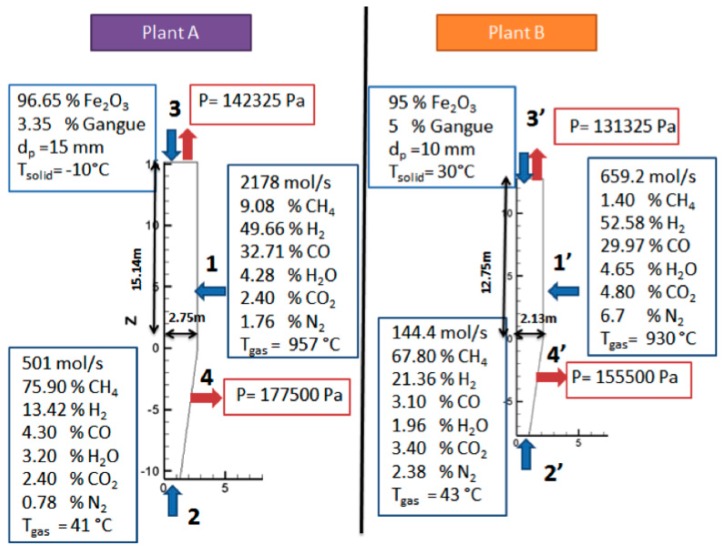
Operating conditions of plants A and B.

For the gas flow, a known pressure condition is also required at the exits. The top pressure was known but not the pressure of the cooling gas outlet, as shown in Figure 3, at Point 4. The latter was estimated to obtain approximately 90% of the inlet cooling gas expelled from this outlet and, approximately, 10% flowing upwards.

Figure 3 shows the values of the known boundary conditions for the two simulations conducted, corresponding to two different plants. Plant A is a North American, MIDREX plant, currently in operation, the main operating data of which were provided to us. Plant B was the first MIDREX plant operated in the USA, for which published data are available [10]. The production capacity of plant A is 4.5 times greater that of plant B.

### 2.6. Meshing and Numerical Solution

**Figure 4:**
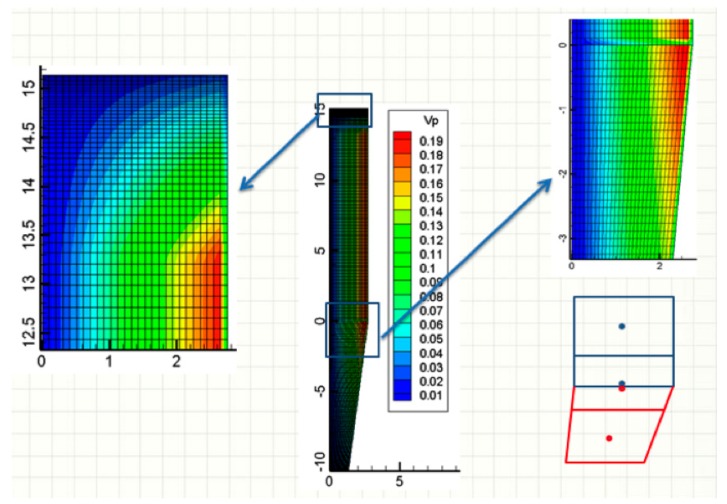
Meshing and volumes (m^3^) of the cells.

The system of partial derivative equations was discretized and solved, according to the finite volume method [28]. Meshing of the cylindrical reduction and transition sections is orthogonal, with cells made finer next to the top, as shown in Figure 4, on the left. For the conical section, a non-orthogonal grid was used, as shown in Figure 4, on the right. To easily connect the two sections, the number of radial cells was kept the same. The numerical code was written in the language of FORTRAN 1995.

## 3. Results and Discussion

In this section, the results of the Plant A simulation are first presented and discussed, then a comparison between the calculated and measured data for both plants is given. Results for the values of the different variables, throughout the reactor, are given in separate figures; however, all of these variables must be considered simultaneously for interpretation purposes.

### 3.1. Pressure Field, Velocity of Gas and Temperature Field

**Figure 5:**
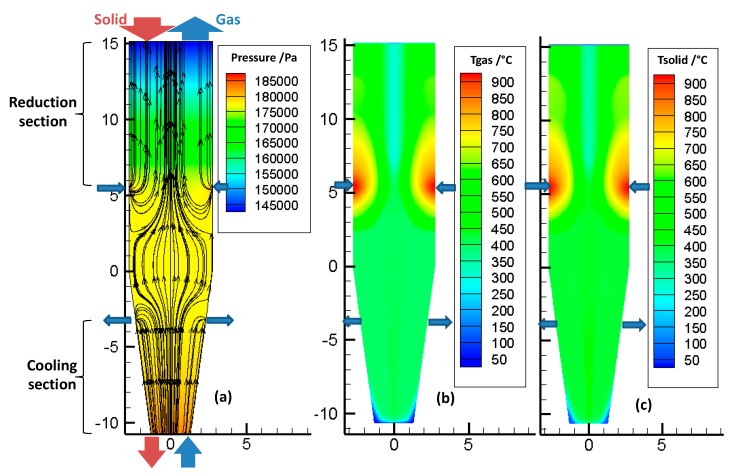
(**a**) Pressure field and velocity streamlines of gas flow inside the bed, (**b**) temperature distribution of the gas phase, and (**c**) temperature distribution of the solid phase.

Figure 5a shows the pressure and velocity fields inside the bed, throughout the reactor. The color scale refers to the pressure, and the lines refer to the streamlines. The large arrows indicate the locations of the various gas and solid inlets and outlets. These locations are the same (and not repeated everywhere) in the following figures. The pressure decreases almost linearly, from bottom to top. The reducing gas, injected at the sidewall (*z* = 5.32 m), enters radially and then flows essentially vertically, except in the transition zone. The cooling gas first flows upwards, and then, most of it leaves the furnace radially, at the cooling gas outlet, except for a fraction that rises in the reduction section.

Figure 5b,c show the temperature distribution of the gas and solid phases in the reactor. First, it was found that the gas and solid temperatures were very close to each other. This similarity resulted from the high gas-to-solid heat transfer, as was described in a previous study [14]. Downwards from the solid inlet, the solid temperature rapidly increased to reach the gas temperature. Second, the temperatures were not axially or radially uniform, throughout the reactor. The hottest zone was near the reducing gas inlet, with gas introduced at 957 °C. Above this inlet, the temperature decreased, because of methane reforming (as shown later, in Figure 7), an endothermic reaction. Third, the cooling gas not only cooled the solid in the bottom section but also influenced the temperature field in the reduction section, with the gas rising from the cooling zone to the central part of the reduction zone. This maintained a lower temperature alongside the center of the shaft.

From these results, radial gradients of temperature were revealed to influence, together with the gas composition profiles, the reduction of the solids and the metallization degree achieved.

### 3.2. Solid Mass Fractions

**Figure 6:**
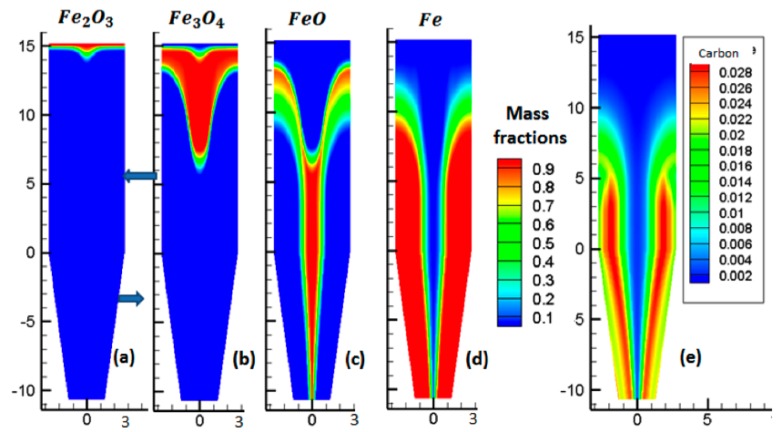
Mass fractions of the solid phases.

Figure 6 plots the evolution of solid mass fractions, throughout the reactor. Figure 6a shows that the hematite was fully converted to magnetite very rapidly in the upper part of the reactor. Subsequently, magnetite was reduced to wüstite, as shown in Figure 6b. Afterwards, wüstite slowly began to reduce to iron, as seen in Figure 6c,d. In the external two-thirds of the reduction section, above the reducing gas inlet—a zone where the gas was rich in H_2_ and CO and the temperature high—the conversion to iron was completed, in approximately 7 m. In the central part of the reactor, where the temperature was lower and the gas, lower in H_2_ and CO, the conversion was not completed and some wüstite remained in the cooling zone. Though the average metallization degree was approximately 94%, metallization was not uniform, with most pellets being completely reduced, whereas others were not.

Figure 6e shows the carbon mass fraction, throughout the reactor. We observed that the carbon was in the same location as Fe, in accordance with the catalytic effect of iron on carbon formation.

### 3.3. Gas Mole Fractions

**Figure 7:**
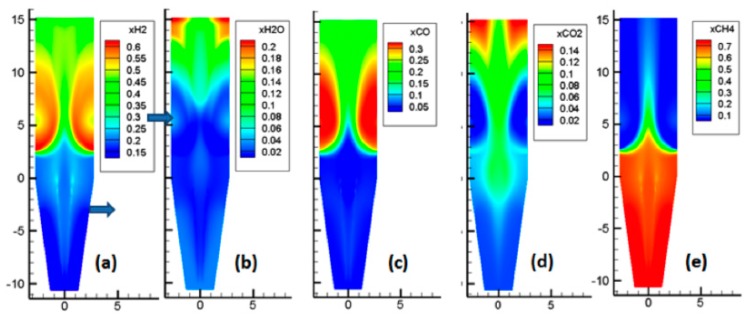
Mole fractions of the gas phase.

As showcased by Figure 7, the situation here is more complex, due to the numerous reactions occurring. The main features of these reactions are as follows. Near the reducing gas inlet, the reforming of methane occurred, which increased the H_2_ and CO contents. Above the gas inlet, the H_2_ and CO contents decreased, while H_2_O and CO_2_ were formed, as a result of the reduction reactions. In the central zone, with less reduction, lower amounts of H_2_O and CO_2_ were formed, and part of the cooling gas, rich in CH_4_, was present.

### 3.4. Overall Picture

**Figure 8:**
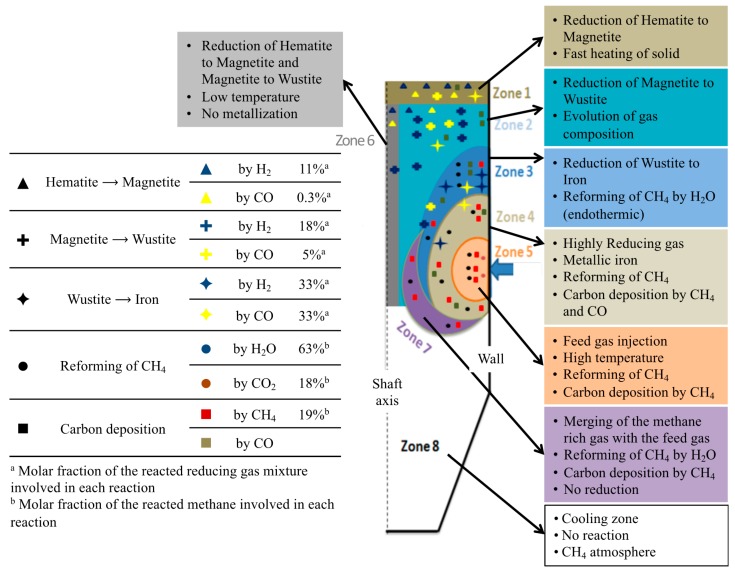
Diagram, illustrating the different zones in the shaft furnace.

Figure 8 is a summary diagram, based on the above results. The shaft furnace was divided into eight zones and distinguished according to the main chemical and thermal processes occurring. On the left part of the diagram are indicated the molar percentages of H_2_ and CO, involved in each reaction, and the molar percentage of methane, reformed by H_2_O or CO_2_, or decomposed to carbon and H_2_. This diagram is an illustration of how modeling work can help one to understand the detailed behavior of a reactor. Clearly, these results could not be obtained from other means.

### 3.5. Validation

Unfortunately, neither interior measurements of solid or gas temperatures, nor compositions, were available for comparison with the calculations. However, from some published data regarding Plant B, and from plant data measurements from Plant A, an overall validation of the model was possible.

Table 4 provides a comparison of the simulation results, with the available plant data. It can be seen that the model reproduced the outlet temperatures and compositions quite satisfactorily. From this strong agreement, obtained by simulations of two plants of differing capacities, the model can be considered validated.

## 4. Conclusions

This article presented the modeling and simulation of an iron ore, direct reduction shaft furnace. We developed a new mathematical model, with the aim of introducing a more-detailed description of the chemical processes, compared to previous studies. The model presented is two-dimensional, describes three sections in the shaft, and accounts for eight heterogeneous and two homogeneous reactions. The model was validated against plant data from two MIDREX plants of notably different capacities. From the analysis of the calculated 2D maps of temperature and composition of the gas and solid phases, it was possible to gain new insights into the interior behavior of the shaft furnace and identify different zones, according to the chemical and thermal phenomena occurring. One significant result is the presence of a central zone of the shaft of lesser temperature and conversion.

Such a model can be helpful in: Investigating the influence of various parameters and operating conditions (including the reducing gas composition), comparing different furnace configurations, and suggesting improvements [29]. These investigations will be the subject of a future paper.

## Figures and Tables

**Table 1:** Source terms for the gas species mass balances.

Species *i*	*S_i_* mol m^−3^ s^−1^
$H_{2}$	$S_{H_{2}}=-v_{1}-\frac{16}{19}v_{2}-v_{3}+3v_{7}+v_{8}+2v_{9}$
CO	$S_{CO}=-v_{4}-\frac{16}{19}v_{5}-v_{6}+v_{7}-v_{8}-2v_{10}$
$H_{2}O$	$S_{H_{2}O}=v_{1}+\frac{16}{19}v_{2}+v_{3}-v_{7}-v_{8}$
${\mathrm{CO}}_{2}$	$S_{CO_{2}}=v_{4}+\frac{16}{19}v_{5}+v_{6}+v_{8}+v_{10}$
${\mathrm{CH}}_{4}$	$S_{CH_{4}}=-v_{7}-v_{9}$

**Table 2:** Source terms for the solid species mass balances.

Species *j*	*S_j_* kg m^−3^ s^−1^
${\mathrm{Fe}}_{2}O_{3}$	$-3M_{{\mathrm{Fe}}_{2}O_{3}}\left(v_{1}+v_{4}\right)$
${\mathrm{Fe}}_{3}O_{4}$	$M_{{\mathrm{Fe}}_{3}O_{4}}\left(2v_{1}-v_{2}+2v_{4}-v_{5}\right)$
${\mathrm{Fe}}_{0.95}O$	$M_{{\mathrm{Fe}}_{0.95}O}\left(\frac{60}{19}v_{2}-v_{3}+\frac{60}{19}v_{5}-v_{6}\right)$
Fe	$0.95M_{\mathrm{Fe}}\left(v_{3}+v_{6}\right)$
C	$M_{c}\left(v_{9}+v_{10}\right)$

**Table 3:** Kinetic constants.

Reactions	Reaction Rate Constants *k_i_*		References
7	$k_{7}=392\exp\left(\frac{6770}{RT}\right)\left({\mathrm{mol}\;\mathrm{cm}}^{-3}s^{-1}\right)$		[25]
8	Fe	$k_{8}=93.3\exp\left(-\frac{7320}{RT}\right)$ $\left({\mathrm{mol}\;\mathrm{cm}}^{-3}s^{-1}\right)$	[25]
	${\mathrm{Fe}}_{0.95}O$	$k_{8}=1.83\times 10^{-5}\exp\left(\frac{7.84}{RT}\right)$ $\left({\mathrm{mol}\;\mathrm{cm}}^{-3}s^{-1}\right)$	[25]
	${\mathrm{Fe}}_{3}O_{4}$	$k_{8}^{\prime }=2.683372\times 10^{5}\exp\left(-\frac{112000}{RT}\right)$ $\left({\mathrm{mol}\;\mathrm{kg}}_{\mathrm{cat}}^{-1}s^{-1}\right)$	[24]
	${\mathrm{Fe}}_{2}O_{3}$	$k_{8}^{\prime }=4.56\times 10^{3}\exp\left(-\frac{88000}{RT}\right)$ $\left({\mathrm{mol}\;\mathrm{kg}}_{\mathrm{cat}}^{-1}s^{-1}\right)$	[24]
9	$k_{9}=16250\exp\left(-\frac{55000}{RT}\right)$ $\left({\mathrm{mol}\;m}^{-3}s^{-1}\right)$		[16,26]
10	$k_{10}=1.8\exp\left(-\frac{27200}{RT}\right)$ $\left({\mathrm{mol}\;m}^{-3}s^{-1}\right)$ $k_{10}^{\prime }=2.2\exp\left(-\frac{8800}{RT}\right)$ $\left({\mathrm{mol}\;m}^{-3}s^{-1}\right)$		[16,26]

**Table 4:** Comparison of the Plant A and Plant B outlet data with the REDUCTOR model calculations.

		Plant A		Plant B		Unit
		Plant Data	Reductor Results	Plant Data	Reductor Results	
**Outlet solid**	**Composition (%)**					
	Fe_2_O_3_	0	0	0	0	wt %
	Fe_3_O_4_	0	0	0	0	wt %
	FeO	7.47	7.1	n.a.	4.3	wt %
	Fe	85.72	85.9	n.a.	87.77	wt %
	C	2	2.2	2	0.91	wt %
	Gangue	4.71	4.8	6.3	7.02	wt %
	**Production**	119.2	119.8	26.4	27.33	t/h
	**Metallization**	93.8	94	93	95.3	%
**Outlet gas**	**Flow rate**	193	200	n.a.	54	kNm^3^/h
	**Composition (%)**					
	H_2_	40.28	40.41	37	37.72	vol %
	CO	19.58	19.89	18.9	20.87	vol %
	H_2_O	19.03	19.52	21.2	20.61	vol %
	CO_2_	17.09	14.69	14.3	13.13	vol %
	CH_4_	2.95	3.91	}8.6	7.67	vol %
	N_2_	1.02	1.55			vol %
	**Temperature**	285	284	n.a.	285	°C

n.a.: not available.

## Appendix A. Notation

**Latin**	
*a_b_*	specific area of the bed (m^2^/m^3^)
*a_c_*	activity of carbon
*c_t_*	total molar concentration of the gas (mol m^−3^)
*c_pg_*	molar specific heat of the gas (J mol^−1^ K^−1^)
*c_ps_*	mass specific heat of the solid (J kg^−1^ K^−1^)
*d_p_*	pellet diameter (m)
*D*	diffusion or dispersion (*D_a_*, *D_r_*) coefficient (m^2^/s)
*h*	heat transfer coefficient (W m^−2^ K^−1^)
*H*	height of the cylindrical section of the shaft (m)
*H_feed gas_*	height of the reducing gas inlet (m)
*H_inf_*	height of the conical section of the shaft (m)
*K_eq_*	equilibrium constant
*K*	permeability coefficient (kg m^−3^ s^−1^)
*k*	mass transfer coefficient, or reaction rate constant
*M*	molar weight (kg mol^−3^)
*p*	gas pressure
*P_i_*	partial pressure of component *i* (bar)
*r*	radius (m)
R	ideal gas constant (J mol^−1^ K^−1^)
*S*	source term
*T*	temperature (K)
*u*	velocity (m s^−1^)
*v*	reaction rate (mol m^−3^ s^−1^)
*w_j_*	mass fraction of solid *j*
*X*	degree of conversion
*x_i_*	molar fraction of *i* in the gas
*z*	height (m)
**Greek**	
*Δ_r_H*	heat of reaction (J mol^−1^)
*ε*	porosity
*τ*	characteristic time (s)
*λ*	thermal conductivity (W m^−1^ K^−1^)
*µ_g_*	viscosity of the gas (Pa s)
*ρ_g_*	mass density of the gas (kg m^−3^)
*ρ_b_*	mass density of the bed (kg $m_{\mathrm{bed}}^{-3}$)
${\tilde{\rho }}_{j}$	molar density of species *j* in the bed (${\mathrm{mol}\;m}_{\mathrm{bed}}^{-3})$
**Subscripts**	
*b*	bed
*c*	catalyst
*cryst*	crystallite
*chem*	chemical
*diff*	diffusional
*interg*	intergranular
*ini*	initial
*intrac*	intra-crystallite
*interc*	inter-crystallite
∞	in the bulk gas
*eff*	effective (for the bed)
*eq*	at equilibrium
*g*	gas
*grain*	grain
*p*	pellet
*r*	radial
*s*	solid
*z*	axial

## Appendix B. Characteristic Times and Reaction Rates

**Table A1:** Kinetic sub-model of a single pellet. Expressions of the characteristic times. *i*: reaction number (see Section 2.1), *k*: H_2_ or CO.

	Hematite → Magnetite	Magnetite → Wüstite	Wüstite → Iron
External transfer	$\tau_{ext,i}=\frac{{\tilde{\rho }}_{Fe_{2}O_{3},ini}d_{p}}{18k_{g}c_{t}\left(x_{k,\infty }-x_{k,eq\left(i\right)}\right)}$	$\tau_{ext,i}=\frac{8{\tilde{\rho }}_{Fe_{3}O_{4},ini}d_{p}}{57k_{g}c_{t}\left(x_{k,\infty }-x_{k,eq\left(i\right)}\right)}$	$\tau_{ext,i}=\frac{{\tilde{\rho }}_{Fe_{0.95}O}d_{p}}{6k_{g}c_{t}\left(x_{k,\infty }-x_{k,eq\left(i\right)}\right)}$
Intergranular diffusion	$\tau_{diff,interg\left(i\right)}=\frac{{\tilde{\rho }}_{Fe_{2}O_{3},ini}{\left(d_{p}\right)}^{2}}{72{\left(D_{k,eff}\right)}_{interg,i}c_{t}\left(x_{k,\infty }-x_{k,eq\left(i\right)}\right)}$	$\tau_{diff,interg\left(i\right)}=\frac{2{\tilde{\rho }}_{Fe_{3}O_{4},ini}{\left(d_{p}\right)}^{2}}{57{\left(D_{k,eff}\right)}_{interg,i}c_{t}\left(x_{k,\infty }-x_{k,eq\left(i\right)}\right)}$	/
Intragranular diffusion	/	$\tau_{diff,intrag\left(i\right)}=\frac{2{\tilde{\rho }}_{Fe_{3}O_{4},ini}{\left(d_{grain,ini}\right)}^{2}}{57{\left(D_{k,eff}\right)}_{intrag,i}c_{t}\left(x_{k,\infty }-x_{k,eq\left(i\right)}\right)}$	/
Inter-crystallite diffusion	/	/	$\tau_{diff,interc\left(i\right)}=\frac{{\tilde{\rho }}_{e_{0.95}O}{\left(d_{p}\right)}^{2}}{24{\left(D_{k,eff}\right)}_{interc,i}c_{t}\left(x_{k,\infty }-x_{k,eq\left(i\right)}\right)}$
Intra-crystallite diffusion (solid phase)	/	/	$\tau_{diff,intrac,\left(i\right)}=\frac{{\tilde{\rho }}_{Fe_{0.95}O}d_{cryst,ini}^{2}}{24D_{sol}\left(c_{ox,eq}-c_{ox,\infty }\right)}$
Chemical reaction	$\tau_{chem,i}=\frac{{\tilde{\rho }}_{Fe_{2}O_{3}}d_{grain,ini}}{6k_{i}c_{t}\left(x_{k,\infty }-\;x_{k,eq\left(i\right)}\right)}$	$\tau_{chem,i}=\frac{{\tilde{\rho }}_{Fe_{3}O_{4}}d_{grain,ini}}{2k_{i}c_{t}\left(x_{k,\infty }-\;x_{k,eq\left(i\right)}\right)}$	$\tau_{chem,i}=\frac{{\tilde{\rho }}_{Fe_{0.95}O}d_{cryst,ini}}{2k_{i}c_{t}\left(x_{k,\infty }-\;x_{k,eq\left(i\right)}\right)}$

**Table A2:** Kinetic sub-model of a single pellet. Expressions of the reaction rates. *i*: reaction number (see Section 2.1).

Reaction *i*	Reaction Rate mol m^−3^ s^−1^
*i* = 1 and 4	$v_{i}=\frac{1}{3}{\tilde{\rho }}_{Fe_{2}O_{3},ini}{\left\{\tau_{ext,i}+2\tau_{diff,interg\left(i\right)}\left[{\left(1-X_{i}\right)}^{-\frac{1}{3}}-1\right]+\frac{\tau_{chem,i}}{3}{\left(1-X_{i}\right)}^{-\frac{2}{3}}\right\}}^{-1}$
*i* = 2 and 5	$v_{i}=\frac{1}{3}{\tilde{\rho }}_{Fe_{2}O_{3},ini}{\left\{\tau_{ext,i}+2\left(\tau_{diff,interg\left(i\right)}+\tau_{diff,intrag\left(i\right)}\right)\left[{\left(1-X_{i}\right)}^{-\frac{1}{3}}-1\right]+\frac{\tau_{chem,i}}{3}{\left(1-X_{i}\right)}^{-\frac{2}{3}}\right\}}^{-1}$
*i* = 3 and 6	$v_{i}=\frac{1}{3}{\tilde{\rho }}_{Fe_{2}O_{3},ini}{\left\{\tau_{ext,i}+2\left(\tau_{diff,interc\left(i\right)}+\tau_{diff,intrac,\left(i\right)}\right)\left[{\left(1-X_{i}\right)}^{-\frac{1}{3}}-1\right]+\frac{\tau_{chem,i}}{3}{\left(1-X_{i}\right)}^{-\frac{2}{3}}\right\}}^{-1}$
